# Supplementary material for: The Cx43 Carboxyl-Terminal Mimetic Peptide αCT1 Protects Endothelial Barrier Function in a ZO1 Binding-Competent Manner
Source: Biomolecules. 2021 Aug 12;11(8):1192. doi: 10.3390/biom11081192 (PMC8393261; doi:10.3390/biom11081192)
Supplement: Supplementary file 1 [file biomolecules-11-01192-s001.zip › biomolecules-1323468-supplementary.pdf]

## Supplementary Materials

A

B

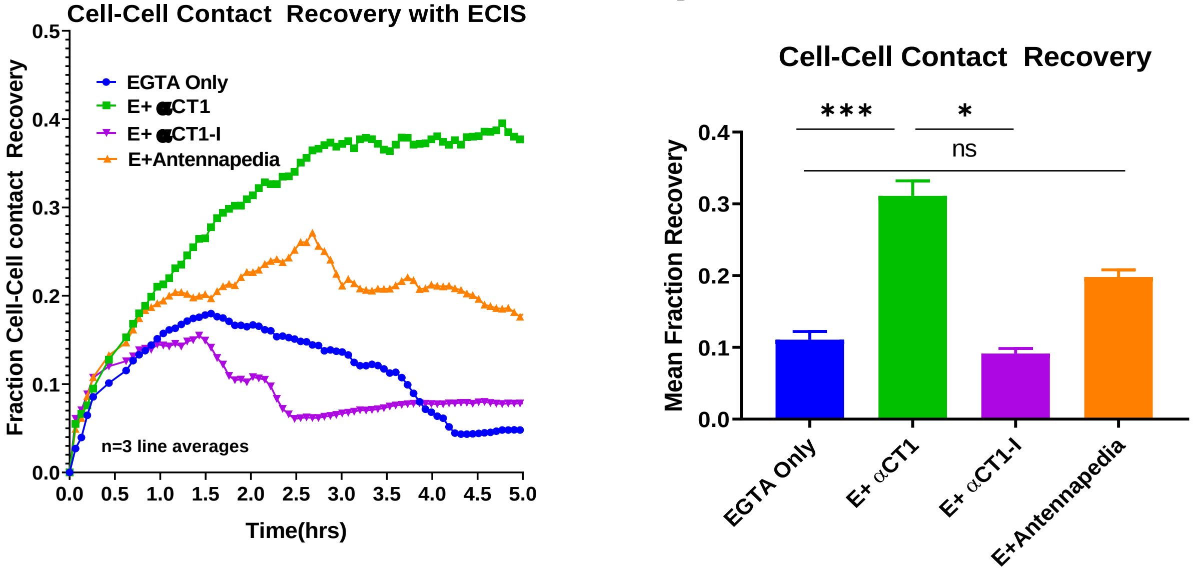


**Figure S1: αCT1 augments barrier function recovery in Cx43-deficient MDCK cells A)** Summary ECIS time course data of barrier function recovery calculated as fraction of barrier function recovery, normalized to the difference between baseline and time points of maximal disruption. **B)** Quantification of area under the curve analysis of barrier function recovery across 5 hour time period, applied to time course data from Figure 4A.

| | | F-actin | VEC |
|---|---|---|---|
| | Cell Location | P<0.05 Vs Thrombin | P<0.05 Vs Thrombin |
| | 1 | V | V |
| Nucleus | 2 | V | V |
| | 3 | V | V |
| | 4 | V | V |
| | 5 | V | V |
| | 6 | V | V |
| | 7 | V | V |
| | 8 | V | V |
| | 9 | V | V |
| Cytoplasm | 10 | V | V |
| | 11 | V | V |
| | 12 | V | V |
| | 13 | V | V |
| | 14 | V | V |
| | 15 | V | V |
| | 16 | V | V |
| Cell Border | 17 | V,A1 | V,A1 |
| | 18 | V,A1 | V,A1 |
| | 19 | V,A1 | V,A1 |
| | 20 | V,A1 | V,A1 |

**Table S1:** Peptide treatments with significant effects compared to thrombin treatment alone in HMEC-1

**V**=Vehicle **A1**=αCT1+T **A-I**= αCT1-I +T

| | | F-actin | ZO1 | Cx43 |
|---|---|---|---|---|
| | Cell Location | P<0.05 Vs Thrombin | P<0.05 Vs Thrombin | P<0.05 Vs Thrombin |
| | **1** | A1 | A1 | A1 |
| Nucleus | **2** | A1 | A1 | A1 |
| | **3** | A1, A-I | A1 | A1, A-I |
| | **4** | V, A1, A-I | A1 | A1, A-I |
| | **5** | V, A1, A-I | A1 | A1, A-I |
| | **6** | V, A1, A-I | A1 | A1, A-I |
| | **7** | V,A1, A-I | A1 | A1, A-I |
| | **8** | V,A1, A-I | A1 | A1, A-I |
| | **9** | V,A1, A-I | A1 | A1, A-I |
| Cytoplasm | **10** | V,A1, A-I | A1 | A1, A-I |
| | **11** | V,A1, A-I | A1 | A1, A-I |
| | **12** | V,A1, A-I | A1 | A1 |
| | **13** | V,A1, A-I | A1 | None |
| | **14** | V,A1 | None | None |
| | **15** | V,A1 | None | None |
| | **16** | V | None | None |
| | **17** | None | None | None |
| Cell Border | **18** | V,A1 | None | None |
| | **19** | V,A1 | A1 | A1 |
| | **20** | V,A1 | A1 | A1 |

**Table S2:** Peptide treatments with significant effects compared to thrombin treatment alone in HDMECs. **V**=Vehicle **A1**=αCT1+T **A-I**= αCT1-I +T **ANT**= ANT +T
